# Molecular Characterization of a Novel *Cathepsin L* in *Macrobrachium nipponense* and Its Function in Ovary Maturation

**DOI:** 10.3389/fendo.2021.816813

**Published:** 2022-01-10

**Authors:** Sufei Jiang, Yiwei Xiong, Wenyi Zhang, Junpeng Zhu, Dan Cheng, Yongsheng Gong, Yan Wu, Hui Qiao, Hongtuo Fu

**Affiliations:** ^1^ Key Laboratory of Freshwater Fisheries and Germplasm Resources Utilization, Ministry of Agriculture, Freshwater Fisheries Research Center, Chinese Academy of Fishery Sciences, Wuxi, China; ^2^ Wuxi Fisheries College, Nanjing Agricultural University, Wuxi, China

**Keywords:** *Macrobrachium nipponense*, *cathepsin L*, ovary maturation, mRNA expression, RNA interference

## Abstract

*Cathepsin L* genes, which belonged to cysteine proteases, were a series of multifunctional protease and played important roles in a lot of pathological and physiological processes. In this study, we analyzed the characteristics a *cathepsin L* (named *Mn-CL2*) in the female oriental river prawn, *Macrobrachium nipponense* which was involved in ovary maturation. The *Mn-CL2* was1,582 bp in length, including a 978 bp open reading frame that encoded 326 amino acids. The *Mn-CL2* was classified into the *cathepsin L* group by phylogenetic analysis. Real-time PCR (qPCR) analysis indicated that *Mn-CL2* was highly expressed in the hepatopancreas and ovaries of female prawns. During the different ovarian stages, *Mn-CL2* expression in the hepatopancreas and ovaries peaked before ovarian maturation. *In situ* hybridization studies revealed that *Mn-CL2* was localized in the oocyte of the ovary. Injection of *Mn-CL2* dsRNA significantly reduced the expression of vitellogenin. Changes in the gonad somatic index also confirmed the inhibitory effects of *Mn-CL2* dsRNA on ovary maturation. These results suggest that *Mn-CL2* has a key role in promoting ovary maturation.

## Introduction

*Macrobrachium nipponense*, also called oriental river prawn, is an important freshwater commercial prawn in China. Female prawns have the characteristics of “Short period of sexual maturity” (1). After entering into the breeding season, females can reach to sexual maturity after being raised for about 45 days, and can reproduce multiple generations in the same year. During the breeding season, especially the water temperature reached above 28°C, it takes only 15 days for females to complete an ovarian maturation cycle (from ovarian emptying to the next spawning). Although it makes an ideal materials for studying crustacean reproduction, however, it has been an important problem for the prawn industry for many years. Frequent reproduction leads to multiple generations living together, causing high breeding density, feed consumption and risk of hypoxia. Therefore, it is very important to find out the key genes of ovary maturation.

The generation and accumulation of yolk is not only a necessary prerequisite for the maturation of oocytes, but also a decisive factor in the female reproductive cycle during the periodic maturation of the ovary of crustaceans (2). Subsequently, we constructed transcriptomes of I–V stages of ovary to detect the key genes that play important roles in the maturation (3). According to the comparison analysis of differentially expressed genes and KEGG enrichment in five stages of ovarian transcriptomes, several genes closely related to rapid ovary maturation were screened. The most significant enriched pathway in vitellogenesis stages was lysosomal pathway and we got several *cathepsin L* genes which might involve in ovary maturation from this pathway (4).

Lysosomal pathway, which contained a variety of hydrolyases, namely, cathepsin, nuclease, phosphatase, glycosidase, lipase, etc., played a main role in decomposing various exogenous and endogenous macromolecules (5). Some lysosomal cathepsin, such as aspartic protease and cysteine proteases identified during ovarian development, were found to be involved in the Vn hydrolysis process of tetrapods and chicken oocytes (6–8). *Cathepsin L*, which belonged to cysteine proteases, was stored in lysosomes as a proenzyme. They were a series of multifunctional protease and played key roles in many pathological and physiological processes (9–11). In mammals, *cathepsin L* participated in not only in proteolysis, but also in antigen presentation, tissue regeneration, metastasis, bone apoptosis, and other important life activities (12–15). So far, *Cathepsin L* genes have been reported in many crustaceans, such as *L. vannamei*, *Metapenaeus ensis*, *Eriocheir sinensis*, *Macrobrachium rosenbergii*, and *M. nipponense* (16–20). These *Cathepsin L* genes all played key roles in immune system of aquatic animal. In insects and fishes, some *Cathepsin L*s were proved to be related to the hydrolysis of vitellogenin (21, 22). The role in reproductive system of *Cathepsin L* were rarely reported in crustaceans.

In this paper, a *cathepsin L2* was characterized from the *M. nipponense* ovary transcriptomes. We analyzed its cDNA sequence and described evolutionary relationship. The expression profiles and tissue location were also processed. An RNAi was used to illustrate the function of *cathepsin L2* in regulating the ovary maturation. The aim of this study is to provide a new idea to solve the “rapid sexual maturation” problem in *M. nipponense*.

## Materials and Methods

### Ethics Approval and Consent to Participate

In this study, no endangered or protected species were involved. The experimental protocols, methods of this study was approved by the Institutional Animal Care and Use Ethics Committee of the Freshwater Fisheries Research Center, Chinese Academy of Fishery Sciences (Wuxi, China).

### Experimental Animals

Adult healthy female *M. nipponense* (weighted 0.73 ± 0.16 g) were collected from the Dapu scientific experimental base of Freshwater Fisheries Research Center (Wuxi, China). All prawns were kept in a recirculating freshwater system under the same environment.

### Sequence Validation and Bioinformatics Analysis

The full-length *cathepsin L2* cDNA sequence of *M. nipponense* was obtained from the transcriptomes of ovary. All sequences of the ovary transcriptome data were deposited in the National Center for Biotechnology Information (NCBI) Sequence Read Archive (accession SAMN11603268-SAMN11603282) under Bioproject PRJNA541783. A pair of primers (listed in **Table 1**) were designed to verify the open reading frame sequence accuracy. Sequence analysis were carried out according to previous research and the phylogenetic tree was performed by MEGA 7.0 using the neighbor-joining method (23).

**Table 1:** Primers used in this study.

Primer	Primer sequence (5’-3’)
*Mn-CL2* F (ORF)	TGTTATGTAGCATAAATTACT
*Mn-CL2* R (ORF)	AAATTCTAGTGACAATGGTCT
*Mn-CL2* F (qPCR)	CGTGTCATCCTTGGAAAGTTTC
*Mn-CL2* R (qPCR)	ATTATTGTCCATGATGTACTGA
*EIF*-F (qPCR)	CATGGATGTACCTGTGGTGAAAC
*EIF*-R (qPCR)	CTGTCAGCAGAAGGTCCTCATTA
*Vg*-F (qPCR)	GAAGTTAGCGGAGATCTGAGGT
*Vg*-R (qPCR)	CCTCGTTGACCAATCTTGAGAG
*Mn-CL2* probe	TCACCTGCTGTTTCTTTGTGTCCCTAGAGAC
*Mn-CL2* anti-probe	GTCTCTAGGGACACAAAGAAACAGCAGGTGA
*Mn-CL2* iF (RNAi)	GATCACTAATACGACTCACTATAGGGTATACAAGAGGGGAGAAAAGA
*Mn-CL2* iR (RNAi)	GATCACTAATACGACTCACTATAGGGAAGAGCTGGGTCTTCACCTGCTG
*GFP* iF (RNAi)	GATCACTAATACGACTCACTATAGGGTCCTGGTCGAGCTGGACGG
*GFP* iR (RNAi)	GATCACTAATACGACTCACTATAGGGCGCTTCTCGTTGGGGTCTTTG

### Spatio-Temporal Expression of *Mn-CL*

qPCR was used to quantify mRNA expression levels of *Mn-CL* with *EIF* gene as the internal reference and the primers were listed in **Table 1** (24). The cerebral ganglion (Cg), eyestalks (E), heart (H), muscles (M), ovaries (O), hepatopancreas (He) and gills (G) were collected from adult female prawns (n = 5). Ovary stages were divided into 5 stages according to previous study (1). Hepatopancreas and ovaries of different ovary stages were collected (n = 5).

### *In Situ* Hybridization (ISH)

The 5 stages ovaries samples of female prawns were collected and fixed in formalin. *In situ* hybridization (ISH) was carried out as reported previously (25). The probes sequences were listed in **Table 1**.

### RNA Interference

The RNAi primers of *Mn-CL* were designed containing T7 promoter (listed in **Table 1**). A *GFP* gene (green fluorescent protein) was selected as a control and its RNAi primers were listed in **Table 1** (26). Ds-RNA of *Mn-CL* and *GFP* were produced by Transcript AidTMT7 High Yield Transcription kit (Fermentas, Inc., USA).

The prawn ovaries of the RNA interference for ovarian development are better to be in the same development period at the beginning of experiment, so that the variation of gonadal development index (GSI) can be counted more easily and visually in the end. At the beginning of this experiment, most female prawns were in ovary stage IV. Therefore, in order to select a large number of females whose ovaries were developing at the same time, stage IV prawns were selected. One hundred healthy female prawns (0.73 ± 0.16 g) in stage IV were randomly divided into two groups and a three-week RNAi experiment was carried out (water temperature was 25 °C). The experiment group (N = 50) was injected with 4 μg/g.b.w of ds-*Mn-CL* each and the injection site is pericardial cavity membrane (25). The same volumes of ds-*GFP* were applied in control group (N = 50). Ds-RNA of *Mn-CL* and *GFP* were injected every five days and five prawns from each group were randomly collected at 1st, 9th, and 17th days after the injection (N =.5). The interference efficiency of was detected in both hepatopancreas and ovary by qPCR. The Vitellogenin (*VG*) mRNA level was also tested to evaluate the regulation of *Mn-CL* on vitellogenin gene. Gonad Somatic Index (GSI = gonadal weight/body weight × 100%) was also calculated to illustrate the role of *Mn-CL* gene in ovarian maturation (1). Ten prawns of the experiment and control groups were randomly sampled on days 1st, 9th, and 17th and the weight of body and ovary were record.

### Expression Detection and Statistical Analysis

The relative expression levels of *Mn-CL*, *Vg* mRNA and *GFP* were calculated using the 2^−ΔΔCT^ method with *EIF* (eukaryotic translation initiation factor 5A) as the reference gene (24, 27). SPSS 23.0 software was used to do statistical analyses and One-way ANOVA and two-tailed t-test were used to analyze statistical differences. All quantitative data described as mean ± standard deviation, and a significant difference was indicated by *P <*0.05.

## Results

### Characterization of Cdna Encoding *Mn-CL2*

**Figure 1:**
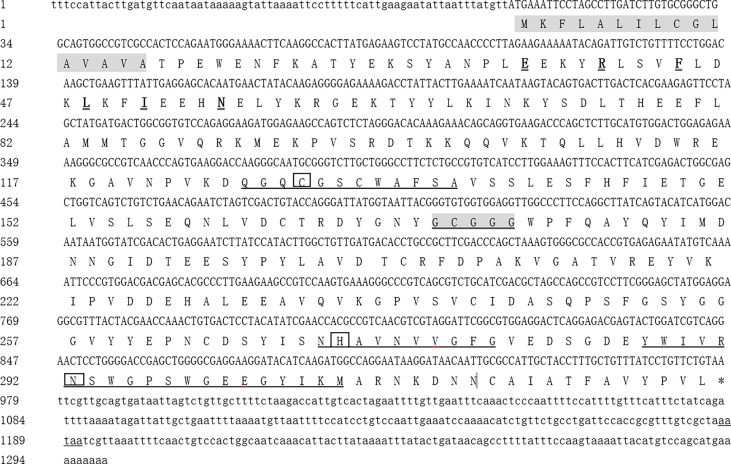
Full cDNA sequence and predicted amino acid sequence of *Mn-CL2* in *M. nipponense*. The shaded area indicates the signal peptide. The black underlined boxes represent the active sites of cysteine, histidine, and asparagine. The *cathepsin L* signature sequences E-X_3_-R-X_2_-I-F-X_3_-N-X_3_-I-X_3_-N were in bold and bigger fonts with double line and GCXGG were shown in shadow underlined. The conserved catalytic triad residues (Cys, His, Asn) are boxed, and its amino sequences are underlined. The asterisk (*) represents the termination codon. The polyadenylation tail signal is underlined.

The *cathepsin L2* of *M. nipponense* were obtained and named *Mn-CL2* (Genbank: OL422141).The *Mn-CL2* gene was1,582 bp long, including a 282 bp and 322 bp of 5′and 3′untranslated region, respectively, and a 978 bp open reading frame (ORF) which encoded a 326 amino acid. The estimated molecular mass was 36.68 kDa. The theoretical pI was 4.65. The *Mn-CL2* pro-peptide contained a 16 amino acid signal peptide and a 310 amino acid mature peptide. Structural prediction analysis indicated three conserved cysteine protease functional sites, a cysteine at 126–137aa, histidine at 270–279aa, and asparagine at 287–306aa (**Figure 1**). *Mn-CL2* belonged to the cysteine protease family of typical cathepsin, containing conserved sequences GCXGG and E-X_3_-I-X_2_-I-F-X_3_-N-X_3_-I-X_3_-N.

### Phylogenetic Analysis and Sequence Alignment of *Mn-CL2*

**Figure 2:**
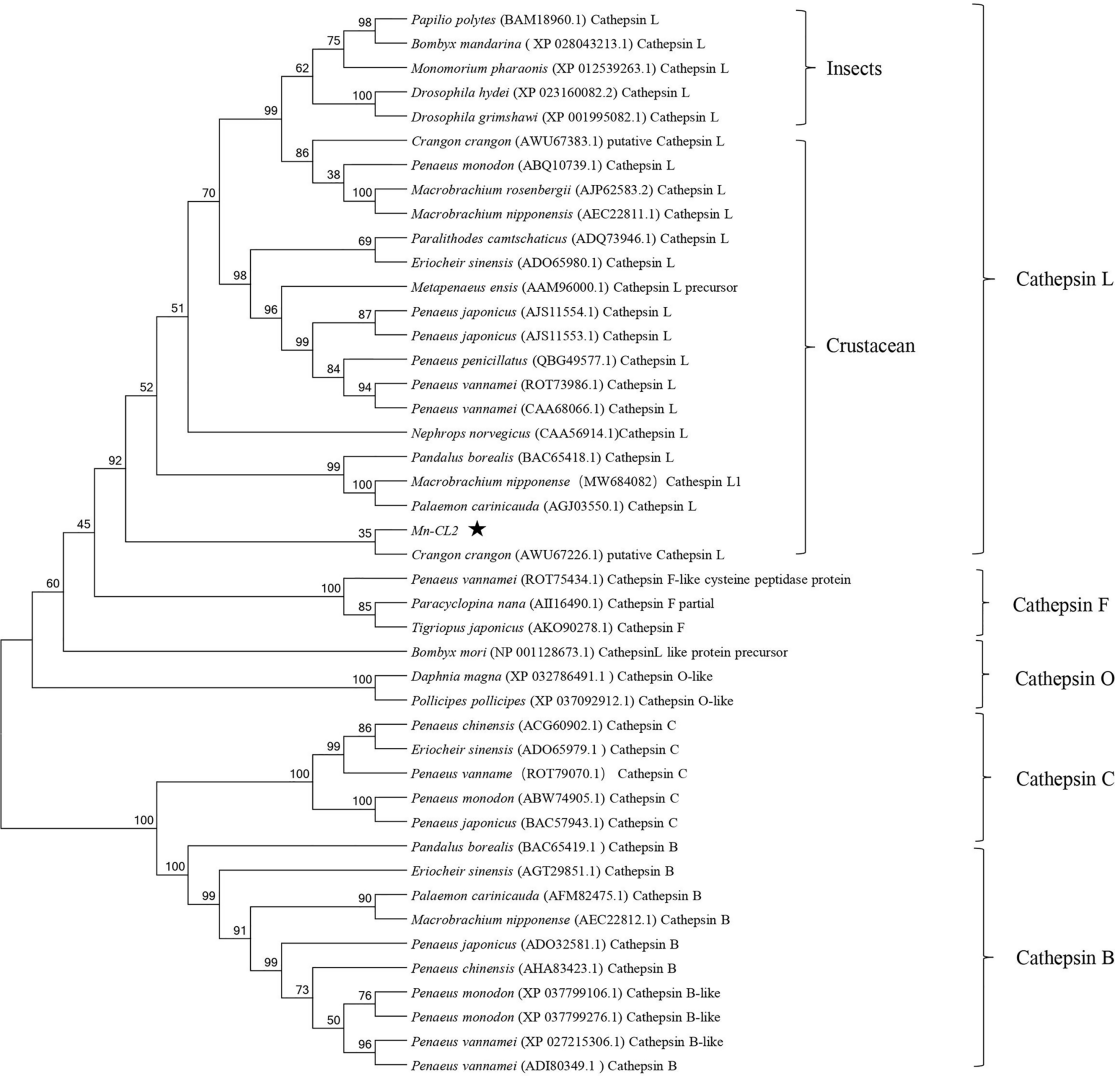
Multiple amino acid alignment and structure prediction of *Mn-CL2* amino acid sequence with *cathepsin L* from other crustaceans. Cathepsin L1 of *M. nipponense*, MW684082; Cathepsin Ls (*M. nipponense*, AEC22811.1; *M. rosenbergii*, AHW49157.1; *Litopenaeus vannamei*, CAA68066; *P. camtschaticus*, AGJ03550.1; *Penaeus japonicus*, AJS11554.1; *Penaeus monodon*, ABQ10739.1; *Eriocheir sinensis*, ADO65980.1). ERFNIN, GCNGG, and GNFD motifs are represented by black boxes. The active sites are marked with red arrows. The symbol star indicated *Mn-CL2*.

Several amino acid sequences of *cathepsin* L, F, O, C, and B in insects and crustaceans were deduced and a phylogenetic tree was built using the NJ method to elucidate the phylogenetic relationships (**Figure 2**). The tree contained two distinct branches, which suggested that *cathepsin* L, F, and O shared common ancestor, as well as cathepsin B and C shared common ancestor. *Cathepsin Ls* were divided into four branches, which do not fully support traditional taxonomic relationships. *Cathepsin L* of *M. nipponense* (AEC22811.1) was clustered with most *Cathepsin Ls* of crustaceans and insects. However, the *Cathepsin L1* (MW684082) and *Mn-CL2* of *M. nipponense*, which obtained from ovary transcriptomes, were clustered in distinct branches, indicating significant differences in structure.

**Figure 3:**
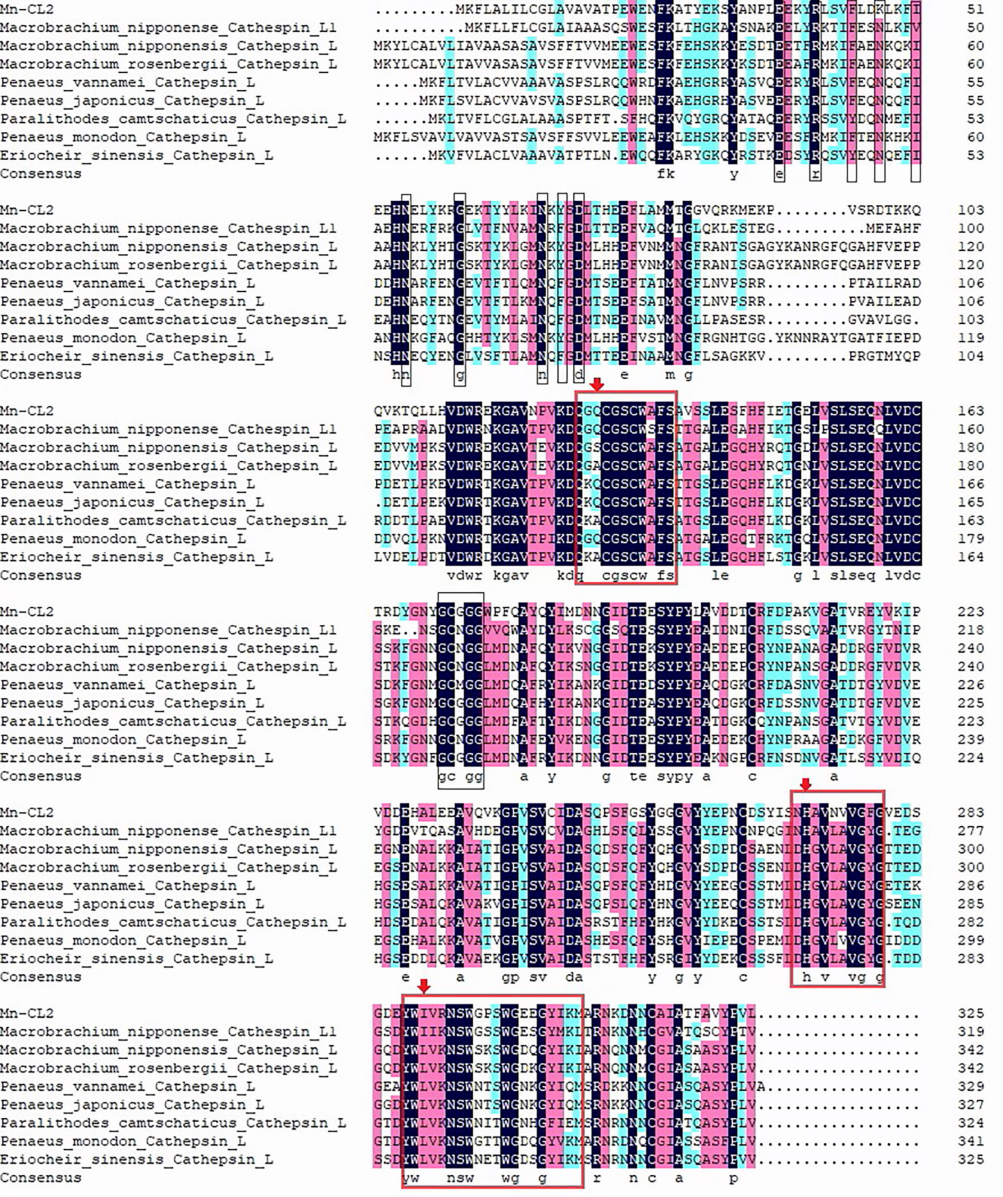
Phylogenetic tree of *Mn-CL2* amino acid sequence in different groups. The numbers below the node indicate the bootstrap value.

Multiple alignments of *Cathepsin L* of crustaceans were built with DNAMAN 6.0 (**Figure 3**).Homology analysis showed that *Mn-CL2* had the highest homology with *Penaeus japonicus* (53.19%). Multiple alignments of *cathepsin Ls* from other crustaceans were performed. The *Mn-CL2* shared amino acid identity with other crustaceans (*M. nipponense*, *M. rosenbergii*, *L. vannamei*, *Paralithodes camtschaticus*, *P. japonicus*, *P. monodon*, and *E. sinensis*) were 45.32, 50.77, 51.37, 50.3, 46.04, and 51.98%, respectively. Comparison of amino acid sequences with other crustaceans showed that the three catalytic active sites of cysteine protease were highly conserved.

### Tissue Distribution and Expression Patterns of *Mn-CL2* in Different Ovary Stages

**Figure 4:**
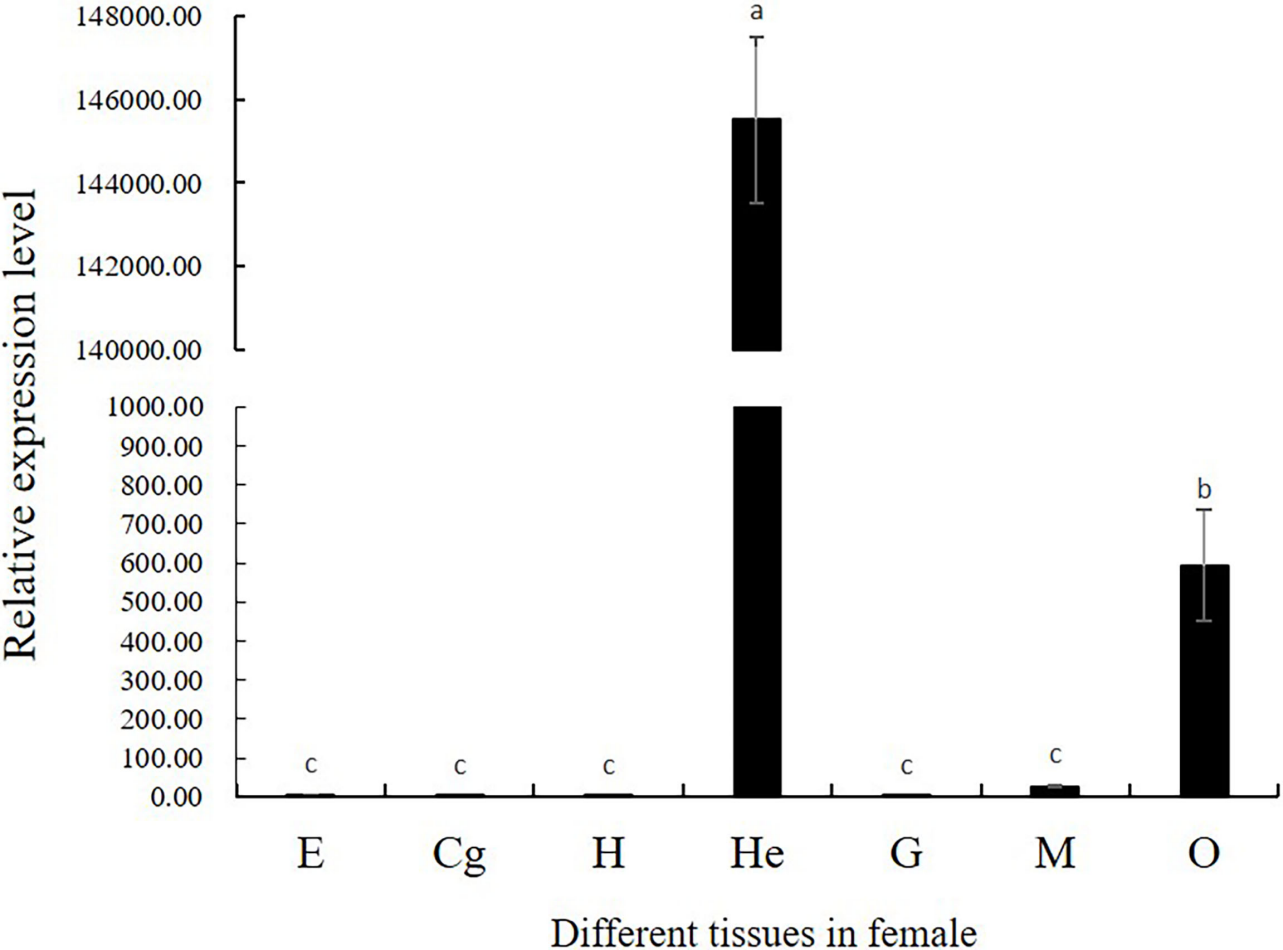
Tissue distribution of *Mn-CL2*. E, eyestalk; Cg, cerebral ganglion; H, heart; He, hepatopancreas; G, gill; M, muscle; O, ovary. Bars with different small letters indicated significant differences (P < 0.05).

The spatio-temporal expression was analyzed in female prawns. Tissue distribution expression was showed in **Figure 4**. The *Mn-CL2* were detected in eyestalks (E), cerebral ganglion (Cg), heart (H), hepatopancreas (He), gills (G), muscles (M), and ovaries (O). The results showed that *Mn-CL2* hardly expressed in cerebral ganglion, heart, eyestalks, and gill. It expressed in muscle weakly. The extremely high expression were detected in hepatopancreas and followed by ovary (*P <*0.01).

**Figure 5:**
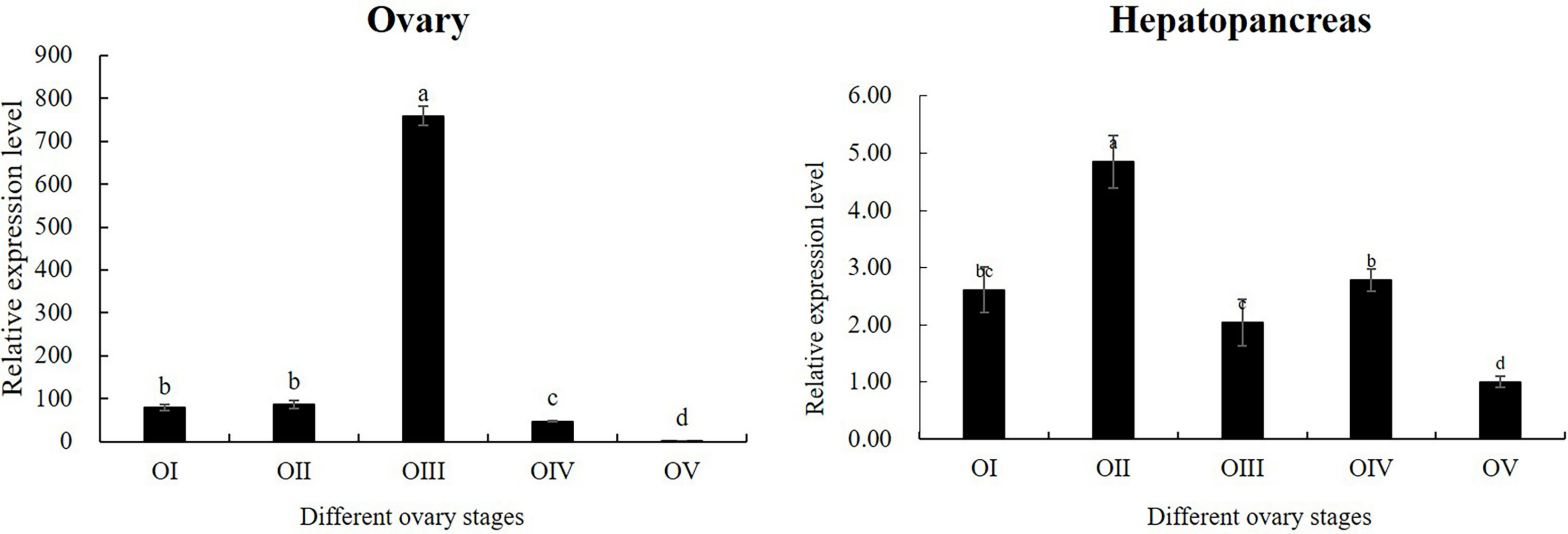
Expression patterns of *Mn-CL2* in the hepatopancreas and ovary at different ovarian stages. Different ovarian stages expressions, OI undeveloped stage, OII developing stage, OIII nearly-ripe stage, OIV ripe stage, OV spent stage. Statistical analyses were performed with one-way ANOVA analysis. Data are shown as mean ± SD (n = 5). Different letters denote significant differences (P < 0.05). Bars with different small letters indicated significant differences (P < 0.05).

According to the results of tissue distribution expression patterns of *Mn-CL2*, we further analyzed *Mn-CL2* expression profiles of hepatopancreas and ovary in five ovarian stages. In ovaries (**Figure 5**), the expression of *Mn-CL2* was relatively stable without significant change from stage I to stage II (*P >*0.05). It increased from stage II to stage III significantly, and the expression level in stage III was 10 times higher than that in stage II (*P <*0.05). Subsequently, its expression dropped dramatically during stage IV, which was 1/15 of what it was at stage III (*P <*0.05). *Mn-CL2* had the lowest expression in ovarian stage V (*P <*0.05). The expression profiles of *Mn-CL2* in hepatopancreas was very different from that in ovary (**Figure 5**). *Mn-CL2* of hepatopancreas maintained a high level expression in each stages of ovary. We found that *Mn-CL2* was relative lower in stages III and V (*P <*0.05) and its expression peaked was detected in stages I and IV (*P >*0.05).

### *Mn-CL2* Localization in the Ovary by ISH

**Figure 6:**
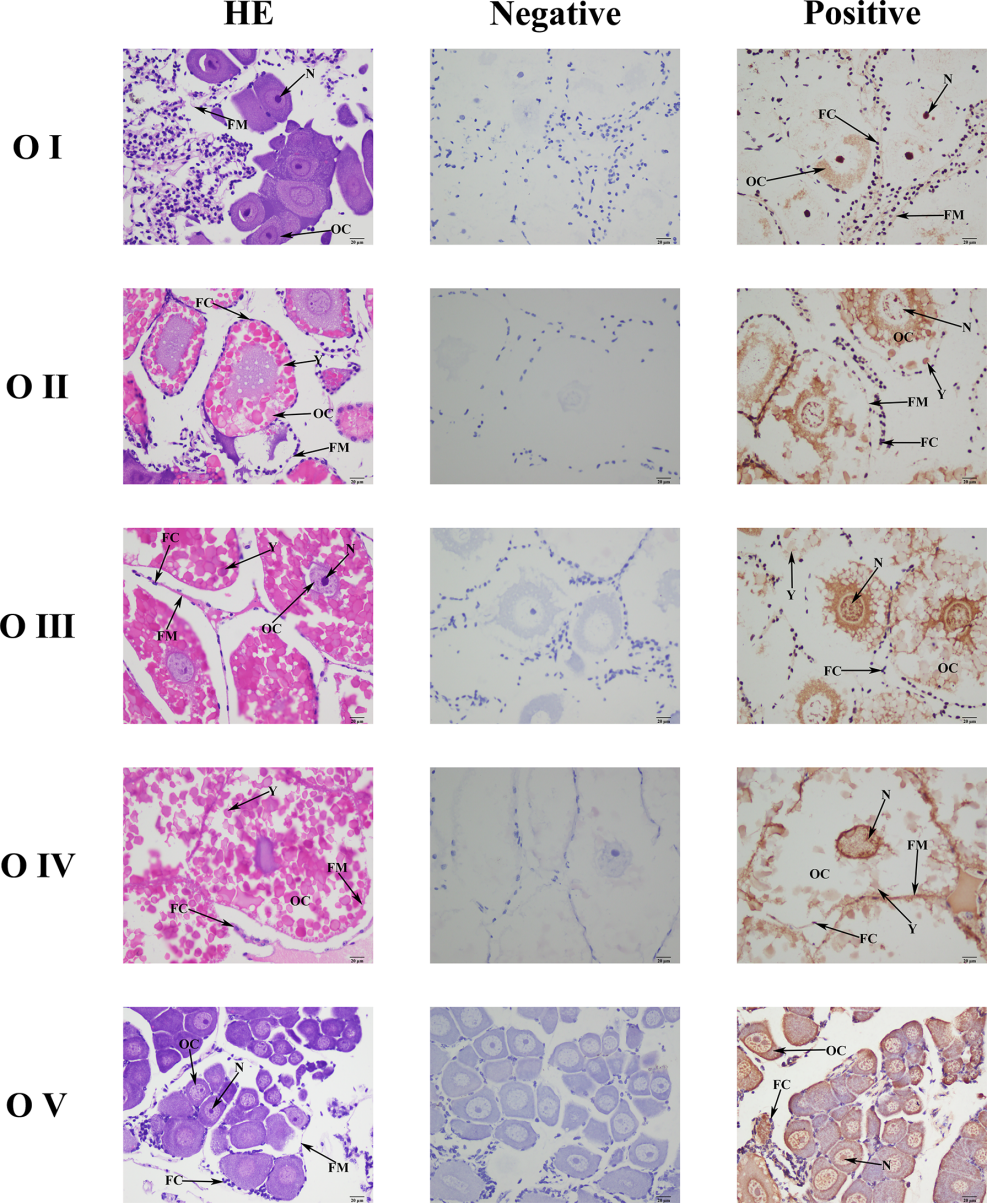
Location of *Mn-CL2* detected in the ovary by *in situ* hybridization. A Photograph of *M. nipponense* ovary in ovarian cycle. OC, oocyte; N, nucleus; CM, cytoplasmic membrane; Y, yolk granule; FC, follicle cell; FM, follicle membrane; Scale bars: ×400.

*Mn-CL2* was detected by ISH in five stages of ovaries to show the cellular localizations (**Figure 6**). The ISH results detected *Mn-CL2* signal was in all ovary stages. The *Mn-CL2* was visualized in oocyte including yolk granule, follicle cell, cytoplasmic membrane, nucleus, and follicle membrane. It mainly expressed in nucleus and follicle membrane. It showed that the signal of Mn-CL2 in the ovary was significantly enhanced during stage II (primary vitellogenesis) to stage III (secondary vitellogenesis), and gradually weakened after stage IV. The signal of Mn-CL2 was weakest in stage I (oogonium proliferation stage).

### The Role of *Mn-CL2* in Ovary Maturation

**Figure 7:**
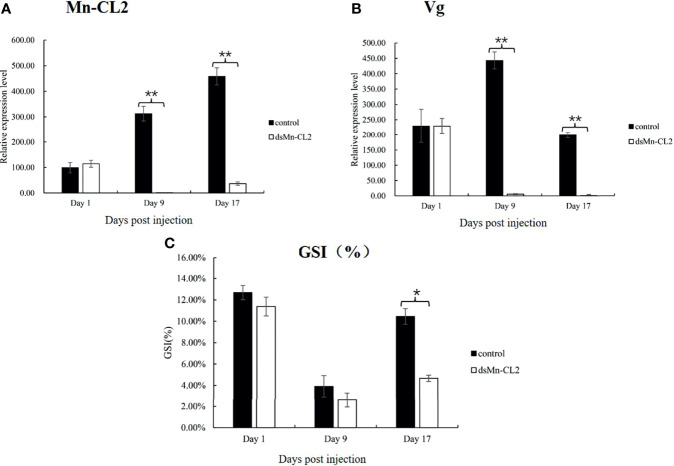
Function analysis of *Mn-CL2* by RNAi. **(A)** Efficiency of RNAi-*Mn-CL2* knockdown in ovary. **(B)** *Vg* transcript after *Mn-CL2* dsRNA injection. **(C)** Changes in GSI (%) of female *M. nipponense* after injection with *Mn-CL2* dsRNA. Data are presented as mean ± SD (n = 6); *denotes statistical significance of P < 0.05. **denotes statistical significance of P < 0.01.

The function of *Mn-CL2* in ovary maturation was revealed by a three-week RNA interference. The results showed that the dsRNA of *Mn-CL2* had a remarkable effect on the ninth day after injection, the expression level of *Mn-CL2* was downregulated remarkably by 99.67% on the 9th day after injection (*P <*0.01) (**Figure 7A**). The *Mn-CL2* expression remained at a very low level which was less than 8% of the control group, till the end of the experiment (on the 17th day). We also tested Vitellogenin (*Vg*) transcript after dsRNA of *Mn-CL2* injection. The *Vg* expression decreased by 98.74% after RNA interference on the 9th day (*P <*0.01). It was almost 200 times higher in the control group than in the interference group (*P <*0.01) at the end of the experiment (**Figure 7B**).

During the interference experiment, GSI (Gonad Somatic Index) was also tracked (**Figure 7C**). Before injection, the average GSI was between 11 and 12% approximately in both control and interference groups while the ovaries of the prawns were almost in stage IV. Later, the ovaries developed from full to empty and enters the next ovarian maturation cycle. The GSI results indicated that on the 1st and 9th days after injection, the ovaries development had no difference between control and interference group (*P >*0.05). At the end of the experiment, the GSI in interference group was only half that of control group (*P <*0.05).

## Discussion

In this study, we identified a novel *cathepsin L* gene from *M. nipponense* named *Mn-CL2*, and put insight into the *Mn-CL2* changes during ovary development, which are the most crucial physiological and morphological processes. *Mn-CL2* was suggested to be involved in a secretary mechanism by containing a typical amino acid signal peptide (28). It belonged to the cysteine family of typical cathepsin, and its precursor domain contains typical *cathepsin L* conserved sequences GCXGG. The 4 amino acids in the GCXGG motif are conserved, while the X represents different amino acids. The X in *cathepsin L* and L1 of *M. nipponense*, *M. rosenbergii*, and *P. monodon* were aspartic acid N, in *M. ensis* is cysteine C, in *L. vannamei* is methionine M and in *E. sinensis* is glycine G. In *Mn-CL2*, The X was glycine G, which was completely different from *cathepsin L* and L1 of *M. nipponense.* It is speculated that the cysteine residues in this motif are located at the corner position and are involved in disulfide bonds formation, which are related to the stability of the enzyme activity region structure and play an important role in the formation of protein structure (29). Therefore *Mn-CL2* may have similar functions and effects of other species *cathepsin L*. Crustacean *cathepsin Ls* contained 4 distinct branches. The high percent identity in residues across taxa indicated that cathepsin L, F and O evolved from gene duplication events (18). Three *cathepsin L* of *M. nipponense* belonged to different branches separately, suggesting the functional variation of *Cathepsin L* in the same species group.

Tissue distribution results proved that the most important tissue for *Mn-CL2* expression is hepatopancreas. Hepatopancreas is the most important site of proteolysis and apoptosis in crustaceans and *cathepsin L* not only participates in proteolysis, but also plays key roles in life activities such as apoptosis, tissue regeneration and antigen presentation (30). This is similar to the results of *cathepsin L* studies in other species (18, 22, 31). In these studies, *cathepsin L* was highly expressed not only in hepatopancreas, but also in brain, heart, gill, hemolymph, and other tissues, which suggested it was closely related to the growth and immune functions. The big difference was that *Mn-CL2* found in this study, as well as *Mn-CTS L1* (another *cathepsin L* detected in the ovary transcriptomes of *M. nipponense* form our lab) expressed highly in ovary. Especially, *Mn-CL2* had rarely expression in other tissues except for hepatopancreas and ovary (4). These results gave evidence that *Mn-CL2* play a special role in the ovaries.

In order to further prove the function of *Mn-CL2* in ovarian development of *M. nipponense*, qPCR was performed in hepatopancreas and ovaries at different ovarian stages. Previous studies (2) suggested that the changes from stages I to II of ovarian development of *M. nipponense* were due to the development of oocytes and cytoplasmic synthesis. It belonged to the early stage of ovarian development. Stages II–III were the rapid ovarian development stage, oocytes continue to grow significantly and yolk granules in the cytoplasm accumulate rapidly. Stage IV was the ovary mature stage in preparing for spawning and Stage V was empty stage after spawning. *Mn-CL2* expression of a marked increase in the amount and reach the maximum, in stage III presumably because Stage III was rapid vitellogenesis stage. It was also a period of rapid growth and proliferation of oocytes, mature egg form requires a lot of yolk proteins provide nutrients and energy. In hepatopancreas, *Mn-CL2* maintained high expression level continuously, suggesting that hepatopancreas is the main production site. Previous studies in freshwater prawn and crab species gave proves that *Vg* was synthesized in hepatopancreas, and then transported to the oocyte through the hemolymph (32, 33). The cellular localizations was detected by ISH and *Mn-CL2* mRNA were examined in all in five ovary stages. We found *Mn-CL2* mRNA signals were stronger in stages II and III which is well distributed in the nucleus and around yolk. *cathepsin L* was reported to be distributed around the cytoplasm and yolk granules in Xenopus (34) which was consistent with the results of this study. All the results indicated that as the lysosome protein, *Mn-CL2* was produced in hepatopancreas and played a key role in ovarian maturation.

The *cathepsin L*, and also B and D, have been proved to play crucial roles in yolk formation in teleosts. In teleosts, vitellogenin (*Vg*) was processed into smaller yolk proteins after it was entered into oocyte by incorporated receptor (22, 35). In the oocyte of marine and freshwater teleosts, the *cathepsin L* and B were involved in yolk proteolysis (36, 37). These cathepsins were supposed to be essential enzymes in yolk compartments mediated by H^+^-ATPase-acidification (38, 39). It was also reported that *cathepsin L* participated in fish and amphibian embryos and larvae yolk protein degradation (40–43). *Cathepsin L* have also been reported in many crustacean species, such as *E. sinensis*, *M. rosenbergii*, and *L. vannamei*, mainly functioned for food digestion and protein degradation to provide energy (17, 18, 44). Researches in other aquatic organisms focuses on physiological processes such as growth, immunity, embryonic development and energy storage (15, 16, 31). In crustaceans, the role of *cathepsin L* involved in ovary maturation were rarely reported.

From the transcriptomes of Stages I–V stages of ovary in *M. nipponense*, we characterized several *cathepsin L* genes which showed evidences that involve in ovary maturation (3, 4) and more details about their roles in crustaceans needs to be clarified and demonstrated. In this paper, RNAi was used to illustrate the special roles of *Mn-CL2* in ovary maturation of *M. nipponense*. RNAi results showed that *Mn-CL2* could downregulated *Vg* expression, which was different from *Mn-CTS L1*. *Vg* is usually expressed in large quantities during specific developmental periods of individuals, and its production, processing and even transportation depend on the regulatory effects of corresponding hormones. We hypothesized that *Mn-CL2* is an important upstream response element of genes involved in regulating ovarian development and *Vg* production. *Mn-CL2* expression in ovary was significantly inhibited from the 9th day after the dsRNA injection and the inhibitory effects lasted until the end of the experiment (17th day). The results proved that ds-*Mn-CL2* was effective and specific for *Mn-CL2* function analysis. The ds-*Mn-CL2* also showed extremely inhibitory effects on *Vg* gene expression suggesting *Mn-CL2* may play an important role in inhibiting production of yolk protein. GSI result directly showed the inhibitory effect of ds-*Mn-CL2* on ovary maturation. At the end of the experiment, the GSI in control group increased almost to the peak, while the GSI in interference group was only half that of control group. All these results suggested that *Mn-CL2* played an important role in yolk generation, and inhibiting the expression of *Mn-CL2* gene can effectively inhibit ovarian maturation.

## Conclusion

In this study, we reported new basic knowledge about a novel *cathepsin L* in crustaceans. The function analysis of *Mn-CL2* showed that inhibiting *Mn-CL2* can effectively inhibit ovarian maturation. Our data provided new information about the regulation of ovarian maturation in *M. nipponense* and may be helpful for solving the problem of rapid *M. nipponense* development in the aquaculture industry. However, the relationship between other regulatory genes and ovarian maturation requires further study.

## Data Availability Statement

The datasets presented in this study can be found in online repositories. The names of the repository/repositories and accession number(s) can be found in the article/supplementary material.

## Ethics Statement

The animal study was reviewed and approved by the Institutional Animal Care and Use Ethics Committee of the Freshwater Fisheries Research Center, Chinese Academy of Fishery Sciences (Wuxi, China).

## Author Contributions

Designed the study, HQ and HF. Carried out the experiments and writing original draft, SJ and WX. Provided technical assistance, WZ and JZ. Methodology and data curation, SJ and WX. Resources, DC and YG. Software, YW. All authors contributed to the article and approved the submitted version.

## Funding

This research was supported by grants from the Jiangsu Agricultural Industry Technology System (JATS [2021] 515); the National Key R&D Program of China (2018YFD0901303); the Central Public-interest Scientific Institution Basal Research Fund CAFS (2020TD36); the China Agriculture Research System-48 (CARS-48); and the New cultivar breeding Major Project of Jiangsu province (PZCZ201745).

## Conflict of Interest

The authors declare that the research was conducted in the absence of any commercial or financial relationships that could be construed as a potential conflict of interest.

## Publisher’s Note

All claims expressed in this article are solely those of the authors and do not necessarily represent those of their affiliated organizations, or those of the publisher, the editors and the reviewers. Any product that may be evaluated in this article, or claim that may be made by its manufacturer, is not guaranteed or endorsed by the publisher.
